# A third-generation bisphosphonate, minodronic acid (YM529), successfully prevented the growth of bladder cancer *in vitro* and *in vivo*

**DOI:** 10.1038/sj.bjc.6603423

**Published:** 2006-10-17

**Authors:** K Sato, T Yuasa, M Nogawa, S Kimura, H Segawa, A Yokota, T Maekawa

**Affiliations:** 1 Department of Transfusion Medicine and Cell Therapy, Kyoto University Hospital, Kyoto 606-8507, Japan; 2 Department of Urology, Akita University School of Medicine, 1-1-1 Hondo, Akita 010-8543, Japan

**Keywords:** bladder cancer, bisphosphonate, minodronic acid, bone metastasis, orthotopic model

## Abstract

Minodronic acid (YM529) is a third-generation bisphosphonate (BP) that has been shown to directly and indirectly prevent proliferation, induce apoptosis, and inhibit metastasis of various types of cancer cells. In this study, we have investigated the therapeutic efficacy of YM529 against bladder cancer, both *in vitro* and *in vivo*. YM529 inhibited geranylgeranylation as well as farnesylation and reduced the growth of all seven bladder cancer cell lines in a dose- and time-dependent manner *in vitro*. YM529 demonstrated a good synergistic or additive antiproliferative effect when administered in combination with cisplatin or paclitaxel. Immunohistochemical study revealed YM529 inhibited the prenylation of Rap1A *in vivo*. YM529 administered systemically did not markedly inhibit the growth of visceral metastases but it showed a significant anticancer effect on bone metastases monitored by an *in vivo* imaging system. Moreover, intravesical YM529 demonstrated significant growth inhibition in a bladder cancer orthotopic model. No adverse effects were associated with the systemic as well as the intravesical treatment regimens. In conclusion, our study suggests that YM529 may be a potent anticancer agent for bladder cancer. The efficacy and safety of this BP as an agent for combination chemotherapies against bladder cancer should be verified by early-phase clinical trials.

Bladder cancer is the fifth most common solid malignancy in the United States. It is estimated that in 2004 there would be 60 240 new cases of and 12 710 deaths from bladder cancer in the United States (Jemal *et al*, 2004). Although 70% of bladder cancers are superficial at presentation and able to be managed by transurethral resection, 60–70% of superficial tumours recur and 20–30% of recurrent disease progresses to higher stages or grades (Messing, 2001; Gee *et al*, 2002). Radical cystectomy is the standard treatment for operable invasive bladder cancer, and the combination of methotrexate, vinblastine, doxorubicin, and cisplatin (MVAC) chemotherapy is the common therapeutic option for distant metastases (Dreicer, 2001; Messing, 2001). The metastatic sites of bladder cancer are lung, liver, and bone in order of preference (Babaian *et al*, 1980). Although bladder cancer is a chemosensitive tumour, most deaths from bladder cancer are caused by invasion and subsequent metastases that are resistant to conventional chemotherapy (Dreicer, 2001; Hussain and James, 2003). In order to improve the outcome for patients with advanced bladder cancer, the development of novel therapeutic options are mandatory.

Bisphosphonate (BP) is an inhibitor of bone-resorption and has been shown to directly and indirectly prevent proliferation, induce apoptosis, and inhibit metastasis of various types of cancer cells (Kuroda *et al*, 2003; Green, 2004; Santini *et al*, 2006). Bisphosphonates inhibit farnesyl pyrophosphate synthase, which is part of the mevalonate pathway. Consequently, BPs inhibit the activation of small G-proteins such as Ras, Rap1, and Rho, and reduce the signals necessary for cancerous progressions, which are transduced by these proteins (Kuroda *et al*, 2003; Nogawa *et al*, 2005b; Sato *et al*, 2005; Yuasa *et al*, 2005).

To date, there has been only one study addressing the possibility of a direct effect of BPs on growth and survival of human bladder cancer cells. Inoue *et al* (2005) demonstrated relatively high concentrated minodronic acid (YM529), which is the most potent third-generation BP (Dunford *et al*, 2001), alone or in combination with docetaxel, reduced the growth of UM-UC-14 bladder cancer cells implanted in tibia (Inoue *et al*, 2005). In order to understand the efficacy of BPs against bladder cancer thoroughly, we investigated the activity of YM529 alone and in combination with cisplatin or paclitaxel on various human bladder cancer cell lines in this study. We have additionally investigated the growth inhibitory effect of YM529 in two different mouse models. First, in order to examine the effect of systemic administration, we used a bone metastatic model, in which cancer cells were inoculated into the left ventricle of the heart and spread throughout the body. Secondly, in order to examine the effect of intravesical administration, we used an orthotopic bladder cancer mouse model.

## MATERIALS AND METHODS

### Animals, cell lines, and reagents

Approval for these studies was obtained from the institutional review board at Kyoto University Hospital. All *in vivo* procedures met the standards required by the United Kingdom coordinating committee on cancer research (UKCCCR) guidelines. In addition, the animal study procedures were consistent and in accordance with the UKCCCR guidelines for the welfare of animals in experimental neoplasia (Workman *et al*, 1998). Pathogen-free 6- to 8-week-old female SCID mice and BALB/c *nu/nu* mice were used in bladder orthotopic and bone metastatic models, respectively (Japan Clea, Osaka, Japan). The human bladder cancer cell lines, 253J, 5637, RT4, RT112, TCCSUP, KU-7, and UM-UC-3, were obtained from the American Type Culture Collection (ATCC, Rockville, MD). UM-UC-3 cell was stably transfected with the pGL3-control vector (Promega, Madison, WI, USA) and with pSV2Neo (ATCC) using Lipofectamine 2000 (Invitrogen, Carlsbad, CA, USA) and named UM-UC-3^LUC^ (Nogawa *et al*, 2005c). YM529, cisplatin, and paclitaxel, were obtained from Yamanouchi (Tokyo, Japan), Nippon Kayaku (Tokyo, Japan), and Bristol–Myers Squibb (New York, NY, USA), respectively.

### Western blot analysis

Protein samples were separated by sodium dodecyl sulfate-polyacrylamide gel electrophoresis and then electroblotted onto a PVDF membrane (Millipore, Tokyo, Japan), as described previously (Kuroda *et al*, 2003). Goat polyclonal anti-unprenylated Rap1A antibody (diluted 1:1000) (Santa Cruz Biotechnologies, Santa Cruz, CA, USA) and mouse monoclonal anti-Ras antibody (diluted 1:1000) (Becton Dickinson, San Jose, CA, USA) were used as the primary antibodies.

### Determination of cell proliferation *in vitro*

Proliferation of the cell lines was measured by Cell Count Reagent SF (Nacalai Tesque, Kyoto, Japan) according to the manufacturer's instructions. Bladder cancer cell lines were cultivated in a flat-bottomed 96-well plate (Greiner Labortechnik, Frickenhausen, Germany) at 3000 cells per well in 100 *μ*l of medium supplemented with 10% fetal bovine serum and incubated with various concentrations of YM529 alone or in combination with cisplatin or paclitaxel for 72 h. The means of six values for each treatment were calculated. For all the cell lines, we evaluated a linear relationship between the degree of proliferation and cell number within the range of the experiment. Half-maximal inhibition constants (IC_50_) were determined using the nonlinear regression program CalcuSyn (Biosoft, Cambridge, UK). To investigate the effect of combining YM529 with other anticancer agents, the cells were treated with six concentrations (0.25, 0.5, 0.75, 1.0, 1.5, or 2.0 × IC_50_) of each agent. Relative cell viabilities indicate the percentile viabilities of each column compared to that of control. To evaluate the combined effects of concurrent treatments, the combination indexes (CI) were calculated using CalcuSyn for Windows software (Chou 1991). This method provides the quantitation of synergism (CI<1) and antagonism (CI>1) at different dose and effect levels. The Fraction affected (Fa) at each dilution was calculated (i.e., Fa of 0.25 would equal 75% viable cells).

### DNA and Annexin V staining

Hoechst 33342 DNA staining (Molecular Probes, Eugene, OR, USA) was performed according to the manufacturer's protocol. To detect apoptotic cells, we performed Annexin V staining using the MEBCYTO Apoptosis Kit (Medical and Biological Laboratories Co., Nagoya, Japan).

### Mouse models

To generate a bone metastatic and an orthotopic models of bladder cancer, UM-UC-3^LUC^ cells were injected into left ventricle (1 × 10^6^/100 *μ*l PBS) and murine bladder (2 × 10^6^/100 *μ*l PBS), respectively, on day 0, as described previously (Nogawa *et al*, 2005a, 2005c). Tumour growth was monitored using an *in vivo* imaging system (IVIS, Xenogen, Alameda, CA, USA) with an aqueous solution of luciferin (150 mg kg^−1^ intraperitoneally), as described previously (Nogawa *et al*, 2005a).

### *In vivo* effects of YM529

#### Bone metastasis model

After injection of UM-UC-3^LUC^ cells, mice were divided into two groups of 10 mice each and the mice were then either left untreated, or were treated with 80 *μ*g kg^−1^ YM529 subcutaneously once a week on days −1, 6, and 13.

#### Orthotopic mouse model

On day 4, the mice were observed by IVIS and the bioluminescence from implanted cancer cells was measured. The mice were then divided into three groups of ten mice in such a way that each group had approximately the same average bioluminescence. The mice were then either left untreated, or were treated five consecutive times from days 5 to 9 with 30 or 100 *μ*M (100 *μ*l) YM529 administered transurethrally.

The bioluminescence from the implanted cancer cells was measured twice a week by IVIS. Three weeks after UM-UC-3^LUC^ cell inoculation, all mice were killed humanely and their sera were collected. The levels of the following serum components were then determined: aspartate aminotransferase (AST), alanine aminotransferase (ALT), lactate dehydrogenase (LDH), total protein (TP), creatinine (Cre), blood urea nitrogen (BUN), and calcium (Ca).

#### Immunohistochemical staining

Immunohistochemical staining was performed by the conventional avidin–biotin–peroxidase complex method (ABC-Elite, Vector Laboratories, Burlingame, CA, USA), as described previously (Nogawa *et al*, 2005c). The orthotopic model mice were either left untreated, or were treated two consecutive times from days 9 to 10 with 30 *μ*M (100 *μ*l) of YM529 administered transurethrally, killed under sufficient anaesthesia, and their bladder were removed at day 10. Goat polyclonal anti-unprenylated Rap1A antibody with a 1:100 dilution was used as the primary antibody. Sections were counterstained with hematoxylin and mounted. Normal mouse IgG was used instead of primary antibody as a negative control.

#### Statistical analysis

The influence of YM529 on the growth of bladder cancers was analysed by the Student's *t*-test using Statmate III (Atoms Co., Tokyo, Japan) statistical software. In all statistical analyses, *P*-values less than 0.05 were judged to be statistically significant.

## RESULTS

### Effect of YM529 on the prenylation of Ras and Rap1A in bladder cancer cells

**Figure 1:**
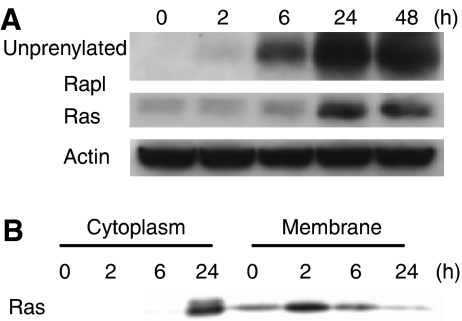
YM529 inhibits both geranylgeranylation and farnesylation *in vitro*. (**A**) Effect of YM529 on the prenylation of Rap1A and Ras in UM-UC-3 cells. The cells were treated with 100 *μ*M YM529 for 2, 6, 24, or 48 h and then lysates were collected and immunoblotted with antibodies specific for unprenylated Rap1A, Ras, or Actin. (**B**) Alteration of cytosolic and membrane-anchored Ras by YM529. UM-UC-3 cells were treated with 100 *μ*M YM529 for 2, 6, or 24 h and cell lysates were collected, separated into cytosolic and membrane fractions, and subjected to Western blotting analysis using an anti-Ras antibody.

To determine whether YM529 inhibits protein geranylgeranylation and/or farnesylation in bladder cancer cells as well as it blocks these events in other cancer cells, we investigated whether YM529 prevents the prenylation of Rap1A (which is activated after geranylgeranylation) and Ras (which is primarily activated after farnesylation). YM529 clearly inhibited the prenylation of Rap1A as it resulted in a time-dependent increase in unprenylated-Rap1A levels (Figure 1A). However, changes in the prenylation status of Ras were less distinct. Consequently, we examined Ras protein levels in cytosolic and membrane fractions by Western blotting. Ras accumulated in the cytosolic fraction and decreased in the membrane fraction after 24 h of YM529 treatment (Figure 1B) suggesting that YM529 inhibits not only geranylgeranylation but also farnesylation in bladder cancer cells. To determine whether the YM529-induced inhibition of prenylation is responsible for the growth inhibitory effect, we investigated whether the YM529-induced growth inhibition was reversed by geranylgeraniol (GGOH), which restores geranylgeranylation, or by farnesol (FOH), which restores farnesylation. However, because cytotoxicity of GGOH and FOH hamper these experiments, we could not determine whether YM529 inhibits geranylgeranylation and/or farnesylation in bladder cancer cells (data not shown). These results are consistent with the previous studies that GGOH and FOH induce apoptosis for the several kinds of cells (Miquel *et al*, 1998, Wright *et al*, 2001).

### Growth inhibition and induction of apoptosis by YM529 *in vitro*

**Figure 2:**
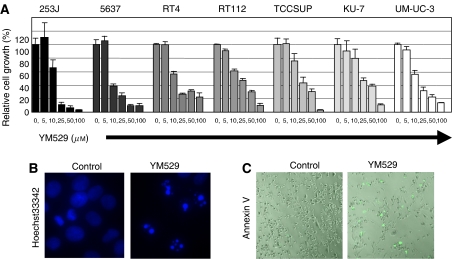
YM529 demonstrates growth inhibition and apoptosis induction of bladder cancer cells *in vitro*. (**A**) Growth inhibitory effect of YM529 alone on bladder cancer cells. Various bladder cancer cell lines were plated at 3000 cells well^−1^ in 96-well plates, incubated for 24 h, and then treated with various doses (0–100 *μ*M) of YM529. After 72 h of incubation, relative cell growth was measured by a modified MTT assay. The data (*n*=6) shown are the means±s.d. (**B** and **C**) Apoptosis induction by YM529 (30 *μ*M, 48 h) was identified by Hoechst 33342 DNA staining (**B**) or FITC-Annexin V staining (**C**).

Next, we investigated the ability of YM529 to inhibit the growth of seven human bladder cancer cell lines using the 3-(4,5-dimethyl-2-thiazolyl)-2,5diphenyl-2H-tetrazolium (MTT) assay. YM529 inhibited the growth of these cells in a dose-dependent manner (Figure 2A). The IC_50_ values of YM529 for growth inhibition in these bladder cancer cell lines, 253J, 5637, RT4, RT112, TCCSUP, KU-7, and UC-UM-3 are 12.2, 7.1, 12.2, 15.4, 21.8, 24.8, and 20.8 *μ*M, respectively. These results indicate that BPs can inhibit the growth of bladder cancer cells, as reported previously for other cancer cells (Kuroda *et al*, 2003; Nogawa *et al*, 2005b; Yuasa *et al*, 2005).

We performed DNA staining of YM529-treated bladder cancer cells to characterize the appearance of the nucleus. Control cells showed a diffuse homogenous staining with Hoechst 33342, but YM529-treated bladder cancer cells showed an abnormal condensed chromosomal appearance, which suggested apoptotic cells (Figure 2B). In order to confirm induction of apoptosis, we investigated Annexin V staining on YM529-treated UM-UC-3 cells. YM529-treated cell cultures contained abundant Annexin V-positive apoptotic cells (Figure 2C), whereas untreated cultures did not. These results indicate that YM529 gave rise to growth inhibition and induced apoptosis in bladder cancer cells *in vitro*. A previous study evaluating the efficacy of zoledronic acid (ZOL), another third-generation BP, in treating osteoporosis found that the peak serum concentrations were in the range of 1–3 *μ*M and were maintained for only a few hours, which indicates that the serum concentrations needed for effective anticancer activity may be difficult to achieve (Berenson *et al*, 2000). However, clinically effective concentrations of YM529 may be readily achieved in two types of cancers, metastatic bone lesions, because of BP accumulation in bone, and orthotopic bladder cancer, because of the feasibility of intravesical administration of YM529 into the bladder.

### Effect of YM529 on bladder cancer growth in a bone metastatic model

**Figure 3:**
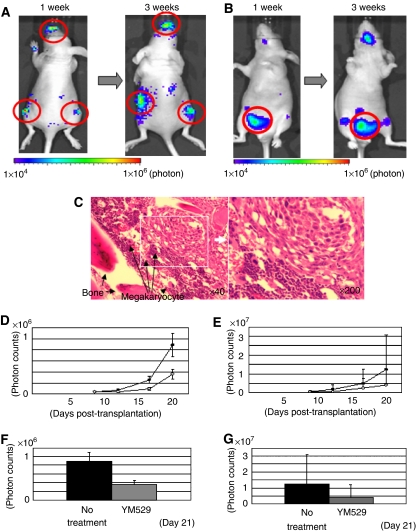
Growth inhibition of bladder cancer by subcutaneous injection of YM529 in a bone metastatic bladder cancer mouse model. (**A** and **B**) To evaluate the effect of YM529 on UM-UC-3^LUC^ cell growth *in vivo*, we selected three metastatic lesions, the maxilla, and hip joints as examples of bone metastasis (**A**) and a lower abdominal lesion as an example of visceral metastasis (**B**), and measured their photon emissions by IVIS. Images were obtained by IVIS at 1 and 3 weeks after transplantation of cells by intracardiac injection. The photon counts of each mouse are indicated by the colour scales. (**C**) Hematoxilin/eosin staining of the bone metastasis of UM-UC-3^LUC^ cells in the bone and bone marrow. (**D** and **E**) Average real-time growth curves of UM-UC-3^LUC^ cells of bone metastatic lesions (**D**, upper) and the visceral metastatic lesion (**E**, upper) in YM529-treated mice and control-untreated mice. Photon emissions of UM-UC-3^LUC^ cells in the mandible and hip joints (**D**, lower) and lower abdomen (**E**, lower) of YM529- treated and untreated mice on day 21 are shown. YM529 significantly prevented the growth of metastatic bone lesions (*P*<0.001), but not of visceral metastasis. Closed circle: no treatment, open circle: YM529 treatment.

*In vivo* implantation of tumour cells transfected with the luciferase gene allows sequential monitoring of tumour growth within the viscera by measuring these photon signals (Adams *et al*, 2002; Hollingshead *et al*, 2004; Uhrbom *et al*, 2004; Nogawa *et al*, 2005a, 2005c). Therefore, we have introduced this quantitative method into this study. Injection of cancer cells via the left ventricle is an established method of inducing bone metastases (Mundy, 2001). When we introduced UM-UC-3^LUC^ cells by intracardiac injection, we found the sites of metastasis and the spreading patterns were reasonably consistent with other cell lines. We monitored the growth of bone metastasis in lesions of maxilla and bilateral hip joints (Figure 3A). Moreover, we evaluated the efficacy of YM529 against visceral metastases by measuring the photon counts of the metastatic lesions in female genitalia and accessory organs (Figure 3B). First, we pathologically confirmed bone metastatic lesion (Figure 3C). The growth of the bone metastatic lesions in YM529-treated mice was significantly less than that of untreated mice (*P*<0.001) (Figure 3D). YM529 showed a tendency to inhibit the growth of visceral metastases, but the effect was not significant (Figure 3E). The body weights and serum components of the two groups of mice did not differ significantly (Table 1). These results suggested YM529 successfully prevented the growth of bone metastases without any adverse side effects, but failed to prevent the growth of visceral metastases. In addition, although we tried to re-estimate the metastatic bone lesions, which we found apparently by IVIS, we could not detect the lesions by CT scan clearly (Supplemental Figure), so we gave up to observe the metastatic bone lesions by CT scan.

### *In vitro* combined effects of YM529 and chemotherapeutic agents

YM529 suppressed bladder cancer cell growth *in vivo*, but did not completely inhibit tumour growth. Our previous studies indicated that BPs augment the effects of several anticancer agents in leukaemia cells (Kimura *et al*, 2004). Therefore, we investigated the use of combinations of YM529 and other chemotherapeutic agents to inhibit the growth of bladder cancer cell lines. Currently, the primary anticancer agent used clinically for bladder cancer is cisplatin and the leading alternative is paclitaxel. The cisplatin and paclitaxel IC50s for cell growth in KU7, RT112, and UM-UC-3 cells were 183 and 0.00207 *μ*M, 266 and 0.00375 *μ*M, and 62.9 and 0.00570 *μ*M, respectively. The combined effects of YM529 and cisplatin or paclitaxel in KU7, RT112, and UM-UC-3 cells are summarised in Table 2. Combination indexes at Fa 0.5 of cisplatin in RT112 and UM-UC-3 and paclitaxel in UM-UC-3 and those at Fa 0.9 of cisplatin in KU7 and RT112 and paclitaxel in RT112 were less than 1.0, indicating synergism. Thus, suggesting that systemic YM529 may be useful for bladder cancer treatment in combination chemotherapy.

### Effect of YM529 on bladder cancer growth in an orthotopic mouse model

**Figure 4:**
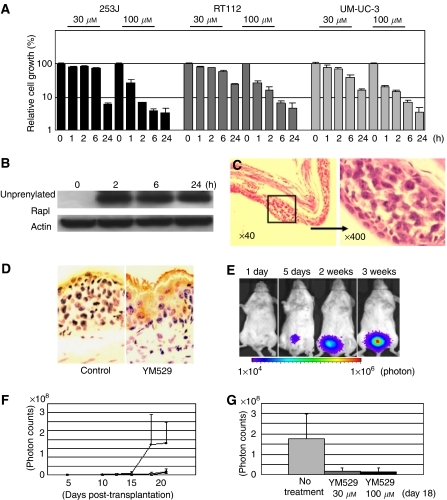
Growth inhibition of bladder cancer by transurethral injection of YM529 in an orthotopic bladder cancer mouse model. (**A**) Comparison of *in vitro* growth inhibition of bladder cancer cells by pulse treatment with YM529 with different treatment times. Relative cell growth was measured 72 h after the initiation of treatments. Bars indicate s.d. (**B**) Alteration of Rap1A prenylation in UM-UC-3 bladder cancer cells by pulse treatment with YM529 for different treatment times. The cells were treated with 100 *μ*M YM529 for 2, 6, or 24 h and then lysates were collected and immunoblotted 24 h after the initiation of treatments. (**C**) Hematoxilin/eosin staining of the transurethral implantation of UM-UC-3^LUC^ cells in the murine bladder. (**D**) Immunohistochemical study revealed unprenylated Rap1A was apparently induced in the cytoplasms of the bladder cancer by intravesical YM529 treatment compared to the control. (**E**) Intravesical growth of bladder cancer in an orthotopic mouse model. Images were obtained by IVIS at 1 day, 1 week, 2 weeks, and 3 weeks following implantation of UM-UC-3^LUC^ cells. The photon counts of each mouse are indicated by the colour scale. (**F**) Average real time growth curves of intravesical UM-UC-3^LUC^ cells in mice treated with 30 or 100 *μ*M YM529 and in untreated mice. Closed circle: no treatment, open circle: YM529 30 *μ*M, closed triangle: YM529 100 *μ*M. (**G**) Photon emissions of intravesical UM-UC-3^LUC^ cells of YM529- treated and untreated mice on day 24 are shown. Transurethral administration of both 30 and 100 *μ*M YM529 prevented the intravesical growth of bladder cancer (*P*<0.01).

YM529 may also be very useful for treatment of bladder cancer, as the drug could be administered directly as an intravesical agent. Before beginning *in vivo* trials, we confirmed that a pulse of YM529 could inhibit growth in cancer cell lines. Relatively high concentrations of YM529 (100 *μ*M) successfully prevented the proliferation of cancer cells even when applied for as little as 1 or 2 h. The relative cell viabilities of 253J, RT112, and UM-UC-3 treated with 100 *μ*M of YM529 for 1 and 2 h, as compared to untreated control cells, were 0.268±0.007 and 0.00717±0.00115, 0.269±0.036 and 0.160±0.037, and 0.203±0.016 and 0.152±0.009, respectively (Figure 4A). We confirmed that a pulse treatment of YM529 successfully prevented the geranylgeranylation of Rap1A in cancer cells, even when applied for as little as 2 h (Figure 4B). The bladder is an ideal target for anticancer agents as they can be given in the high doses often required to produce local cellular effects, with little risk of systemic toxicity. In order to investigate the effect of intravesical YM529 delivery, we used an orthotopic mouse model in which 2 × 10^5^ UM-UC-3^LUC^ cells were implanted into the bladder, as described previously (Nogawa *et al*, 2005c). First we pathologically confirmed the successful implantation of UM-UC-3^LUC^ bladder cancer cells in murine bladder (Figure 4C). Then, we investigated the alteration of Rap1A status in bladder cancer cells by YM529. Immunohistochemical staining of unprenylated Rap1A was apparently induced by intravesical YM529 treatment compared to the control (Figure 4D). These results suggested transurethrally administered YM529 inhibited the prenylation of Ras-related proteins and the signals they mediated in the bladder cancer cells. After the implantation, bioluminescence was not detectable on the day following implantation, but was detected 5 days later, and then increased substantially for up to 3 weeks (Figure 4E). Using this orthotopic mouse model, we investigated the efficacy of intravesical YM529 against bladder cancer. We introduced 30 or 100 *μ*M of YM529 (100 *μ*l) intravesically, and both significantly inhibited bladder tumour cell growth (Figure 4F), although two of 10 mice from the 100 *μ*M YM529-treated group died shortly after intravesical YM529 was administered. Figure 4G demonstrates that by day 24, the inhibitory effects of both 30 and 100 *μ*M YM529 were statistically significant compared to the untreated mice (*P*<0.01). Although a pulse treatment of 30 *μ*M YM529 could not inhibit growth in cancer cell lines *in vitro*, daily intravesical treatment (from days 5 to 9, five times) of YM529 gave rise to growth inhibition of the bladder cancer in the lower dose treatment.

To determine the side effects of these treatments, we examined body weight during the treatment period and the serum concentrations of AST, ALT, LDH, TP, Cre, BUN, and Ca on day 21 after cell implantation. We did not detect any adverse effect on body weight or the serum components tested (Table 1). We assumed that the two mice that died suffered from mechanically induced renal toxicity owing to the implanted bladder cancer, and not a chemically induced side effect of YM529.

## DISCUSSION

This study demonstrates that a third-generation BP, YM529, has a direct effect on the *in vitro* proliferation of bladder cancer cells and on bone metastases and orthotopic bladder cancer *in vivo*. Currently, the systemic combination chemotherapy MVAC is the standard therapy and results in long-term survival in some patients with advanced metastatic disease, although the survival rates are limited. To improve the current therapies for these cancers, clinical trials with several new promising agents have been undertaken. Among these new agents, paclitaxel is the most active single agent in advanced urothelial cancer. A Phase II study of paclitaxel and cisplatin for advanced urothelial cancer demonstrated partial regression in 38% of patients and complete regression in 32% with no major side effects (Burch *et al*, 2000). However, visceral metastases including bone metastases were predictors of a poor prognosis (Bellmunt *et al*, 2002).

BPs are well-known agents that significantly reduce adverse skeletal events including pathologic fracture, spinal cord compression, hypocalcaemia, and severe pain in patients with various malignancies (Ibrahim *et al*, 2003; Lipton *et al*, 2004; Saad *et al*, 2004). On the basis of results from three large, randomised, Phase III clinical trials enrolling more than 3000 patients, ZOL (4 mg via 15-min infusion) was approved in the United States for treatment of patients with documented bone metastases from solid tumours in conjunction with standard antineoplastic therapy (United States Food and drug administration, 2002).

BPs inhibit protein prenylation and demonstrate direct anticancer effects in various cancer cell lines (Green, 2004; Santini *et al*, 2006). Although the clinical effects of BPs against bladder cancer has not been reported, recent report demonstrated that a patient with an acute panmyelosis or bone metastasis of renal cell cancer obtained complete remission with intravenous administrated BP alone (Espanol *et al*, 2004; Michaelson *et al*, 2004), suggesting its potential role as an anticancer agent. Here, we found that YM529 blocks the prenylation of Rap1A and Ras in bladder cancer cells, inhibits the growth of bladder cancer cells *in vitro* (Figures 1A, B and 2A), in a dose- and time-dependent manner, and clearly induces apoptosis in bladder cancer cells (Figure 2B). In the study of leukaemia, we demonstrated that the efficacy and mechanism of action of the anticancer agents YM529 and ZOL were comparable (Segawa *et al*, 2005). These results suggested that third-generation BPs including YM529 and ZOL may be potent antibladder cancer agents.

Consequently, we investigated the growth inhibitory effect of YM529 in a mouse model, namely, a left-ventricular implanted bone metastatic model. Despite the marginal growth inhibition of visceral metastases, treatment with YM529 had a significant antiproliferative effect against bone metastases in this model (Figure 3E and F). The low efficacy of YM529 against visceral metastases may reflect the fact that it is difficult to achieve therapeutically effective serum concentrations (1–3 *μ*M) of YM529 *in vivo*. However, BP has a high affinity for mineralised bone and rapidly localizes to the bones. The concentration of BP in bone tissue reached as high as 800 *μ*M in osteoclast bone (Sato *et al*, 1991). Thus, YM529 may directly promote apoptosis in bone metastatic tumour cells. Although we have not directly measured YM529 concentrations in bone and bone marrow, YM529 clearly has anticancer effects *in vivo*.

Another possible mechanism of bone metastatic model is the indirect *in vivo* growth inhibition through the inhibition of osteoclastgenesis. Bisphosphonates exhibit a high affinity for calcified matrices such as hydroxyapatite in bone, and promote the induction of apoptosis in osteoclasts (Hughes *et al*, 1995). Bone is an abundant repository for immobilized growth factors, including transforming growth factor *β*, fibroblast growth factor, insulin-like growth factors I and II, platelet-derived growth factor, and bone morphogenic proteins (Roodman, 2004). When the osteoclasts absorb bone by secreting proteases, these growth factors are released and provide fertile ground in which tumour cells can grow. Thus, BPs can reduce bone resorption mediated by osteoclast and make a less favourable site for tumour cell growth.

It is critical to know whether the levels that were achieved in these animals are ever likely to be achievable in humans. In orthotopic model, the intravesical concentration would be clearly achievable and the inhibitory mechanism of vesico-ureteral reflux can be worth inhibiting renal toxicity. In bone metastatic model, the concentration of BPs in bone tissue also would be achievable because the concentration of BP in bone tissue reached as high as 800 *μ*M in osteoclast bone (Sato *et al*, 1991). Furthermore, the dosage of YM529 that we injected subcutaneously (80 *μ*g kg^−1^ week^−1^ for 3 weeks) was comparable to the dosage used in Phase I clinical study. Peroral 9 mg of YM529, whose bioavailability was estimated approximately 1%, was administrated to the patients with multiple myeloma daily. Moreover, the concentration that we used was one-third the concentration of the previous renal cancer model. No harmful effects were detected in the renal cancers models (Yuasa *et al*, 2005), nor were adverse reactions apparent in the current study (Table 1). Inoue *et al* (2005) found the growth inhibitory effect of YM529 on UM-UC-14 bladder cancer cells implanted in tibia. They administered YM529 (0.3 mg kg^−1^), once a week for 4 weeks intraperitoneally. The total dose they used are five times more than ours. Thus, the dosage we used may be safe and likely to be achievable in the patients with bladder cancer as well.

Mortality in most cancer patients is increasingly linked to metastatic disease. Bone is the third most common metastatic site of bladder cancer (Messing, 2001), and is infrequently cured by current chemotherapeutic regimens. We investigated YM529 as a therapeutic partner for cisplatin and paclitaxel because these two agents are the central agents in combination chemotherapies for bladder cancer (Burch *et al*, 2000; Dreicer, 2001). YM529 demonstrated good synergistic or additive antiproliferative effects when administered in combination with cisplatin or paclitaxel on three human bladder cancer cell lines (Table 2). These results are consistent with many other studies against a range of tumour cell lines (Jagdev *et al*, 2001; Kimura *et al*, 2004; Matsumoto *et al*, 2005). We speculate that addition of BPs to existing chemotherapeutic regimens, as exemplified by MVAC plus ZOL therapy, might improve the effectiveness of the treatment.

Finally, we investigated intravesical administration against an orthotopic bladder cancer model, as local administration of YM529 in the restricted environment of the bladder could provide a high concentration exposure of the agent to the target cancer cells over a limited time. High concentrations of YM529 had significant anticancerous effects with a pulse treatment (Figure 4A and B). Intravesical Bacillus Calmette-Guerin (BCG) treatment, which is currently the most effective therapy for bladder cancer treatment, causes irritative voiding symptoms in 90% of cases owing to inflammation, which may be necessary for the effective action of this agent. Moreover, BCG sepsis, although rare (0–4%), is a life-threatening condition (Messing, 2001). Options are especially needed for patients who are refractory to intravesical BCG. In this study, we demonstrated that intravesical YM529 (both 30 and 100 *μ*M) successfully prevented growth of bladder cancers (Figure 4F and G), thus demonstrating its effectiveness as a potent chemotherapeutic agent.

In conclusion, YM529 is a third-generation BP with anticancer activity against bladder cancer both *in vitro* and *in vivo*. YM529 demonstrated good synergistic or additive antiproliferative effects when administered in combination with cisplatin and paclitaxel. This indicates that YM529 may be a promising therapeutic strategy for bladder cancer. Recently, the US Food and Drug Administration approved several BPs to treat not only osteoporosis, but also cancer-related bone complications (United States Food and drug administration, 2002). The efficacy and safety of BP as an agent for combination chemotherapy against bladder cancer should be verified by early-phase clinical trials.

## Figures and Tables

**Table 1:** The serum variables on day 21 after cell implantation

**Group**	**AST (IU l^−1^)**	**ALT (IU l^−1^)**	**LDH (IU l^−1^)**	**TP (g dl^−1^)**	**BUN (mg dl^−1^)**	**Cre (mg dl^−1^)**	**Ca (mg dl^−1^)**
*Bone metastasis model*							
Control	95.0±24.6	23.8±9.6	868.9±213.6	4.79±0.24	41.8±20.9	0.14±0.02	9.8±0.4
YM529	70.9±7.7	21.1±4.1	562.5±89.6	5.14±0.31	31.1±1.7	0.11±0.01	10.1±0.3
							
*Bladder orthotopic model*							
Control	110.6±28.0	23.5±3.5	663.3±146.0	4.81±0.81	29.4±3.88	0.08±0.01	10.1±0.4
YM529 30 *μ*M	108.6±22.1	19.6±4.7	653.8±131.4	5.18±0.09	30.5±6.31	0.08±0.01	9.7±0.2
YM529 100 *μ*M	95.8±16.3	23.5±3.8	560.7±112.1	4.96±0.39	33.2±3.60	0.08±0.01	9.6±0.4

Abbreviations: ALT=alanine aminotransferase; AST=aspartate aminotransferase; BUN=blood urea nitrogen; Ca=calcium; Cre=creatinine; LDH=lactate dehydrogenase; TP=total protein.

**Table 2:** CI at Fa of 0.5 and 0.9 of YM529 in combination with cisplatin and paclitaxel in bladder cancer cells

**Cell**	**Agents**	**CI at Fa 0.5**	**Effect**	**CI at Fa 0.9**	**Effect**
KU-7					
	Cisplatin	1.107	Additive	0.696	Synergistic
	Paclitaxel	1.066	Additive	0.914	Synergistic
					
RT112					
	Cisplatin	0.736	Synergistic	0.794	Synergistic
	Paclitaxel	0.844	Additive	1.227	Additive
					
UM-UC-3					
	Cisplatin	0.878	Synergistic	1.044	Additive
	Paclitaxel	0.555	Synergistic	1.135	Additive

CI=Combination indexes; Fa=fraction affected.
