# Supplementary material for: A third-generation bisphosphonate, minodronic acid (YM529), successfully prevented the growth of bladder cancer in vitro and in vivo
Source: Br J Cancer. 2006 Oct 17;95(10):1354–61. doi: 10.1038/sj.bjc.6603423 (PMC2360606; doi:10.1038/sj.bjc.6603423)
Supplement: Supplemental Figure [file 95-6603423x1.doc]

**Supplementary Figure**

**Examination of bone metastasis by IVIS, CT scan, and histology.** (A, B, C)

To evaluate the bone metastatic lesions, we used CT scan, IVIS, and histological examination. We clearly detected the bioluminescence from cancer cells on a left thigh bone by IVIS (A). By histological examination, we could confirm the invasion of UM-UC-3^Luc^ cells (B) (closed arrow: invaded cancer cells, open arrow: normal haematopoietic cells, HE stain, x 100). Despite of careful investigation of a left thigh bone by 0.5mm slice CT scan, we could not detect clearly bone destructions or invasion of cancer cells (C).

#
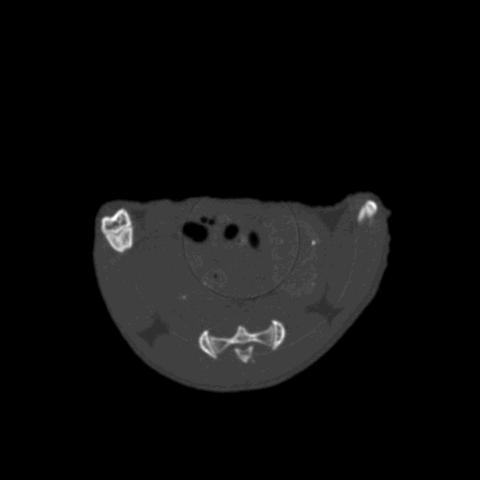

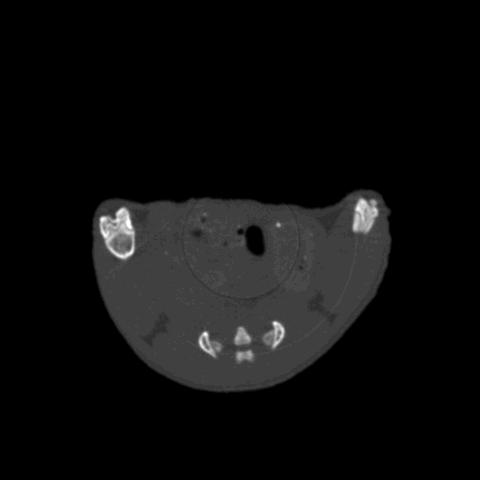

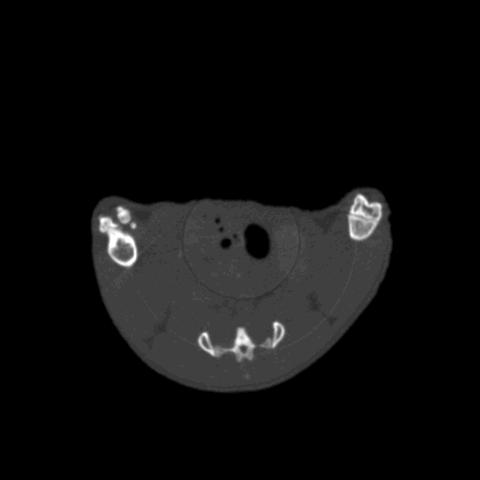

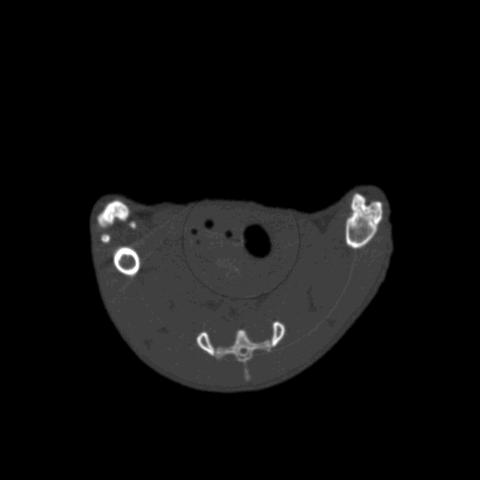

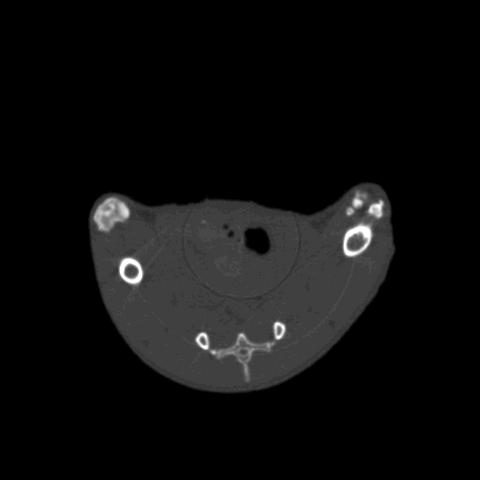

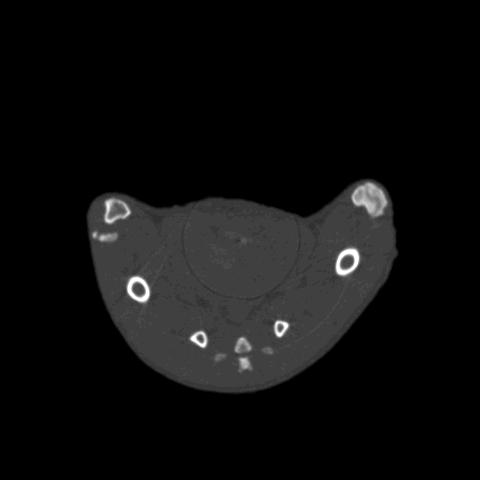

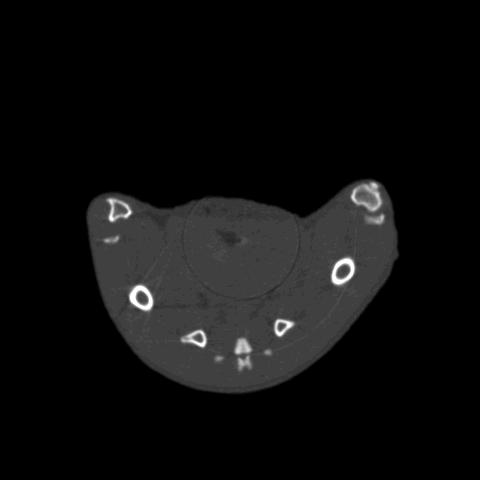

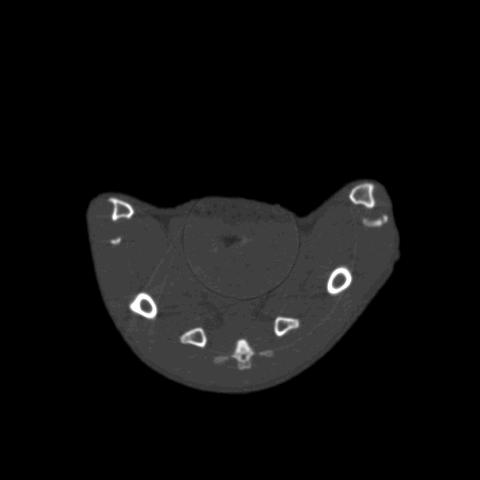

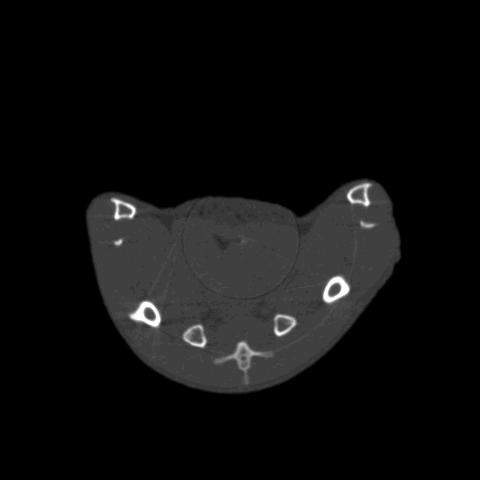

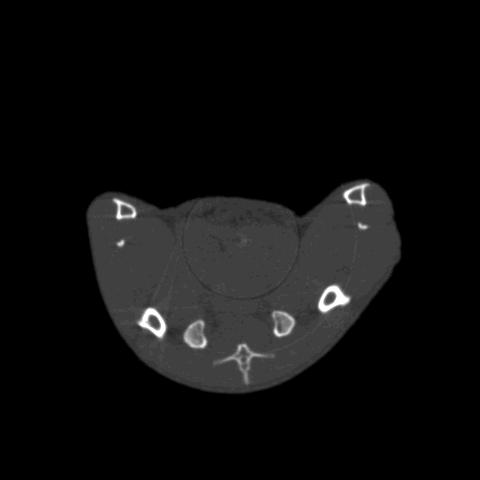

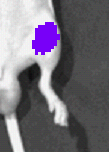

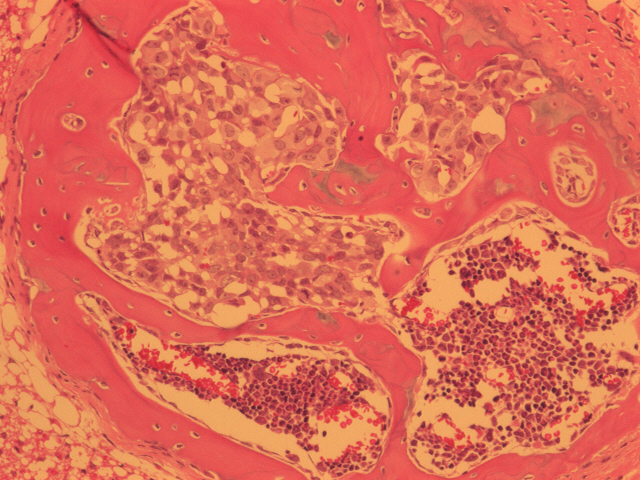
Supplemental Figure

1

2

3

4

5

6

7

8

9

10

**1**

**10**
